# Qualität der dermatologischen Versorgung von Neurodermitis in Deutschland – keine Verbesserung der Indikatoren nach 10 Jahren

**DOI:** 10.1007/s00105-021-04885-3

**Published:** 2021-09-20

**Authors:** A. Langenbruch, N. Mohr, F. Abeck, J. Schmitt, S. Ständer, T. Werfel, D. Thaçi, S. Weidinger, M. Augustin

**Affiliations:** 1 Institut für Versorgungsforschung in der Dermatologie und bei Pflegeberufen (IVDP), Universitätsklinikum Hamburg-Eppendorf (UKE), Martinistr. 52, 20246 Hamburg, Deutschland; 2 Zentrum für evidenzbasierte Gesundheitsversorgung, Medizinische Fakultät Carl Gustav Carus, Technische Universität Dresden, Dresden, Deutschland; 3 Klinik für Hautkrankheiten, Kompetenzzentrum Chronischer Pruritus, Universitätsklinikum Münster, Münster, Deutschland; 4 Klinik für Dermatologie, Allergologie und Venerologie, Medizinische Hochschule Hannover, Hannover, Deutschland; 5 Institut für Entzündungsmedizin, Universitätsklinikum Schleswig-Holstein Campus Lübeck, Lübeck, Deutschland; 6 Klinik für Dermatologie, Venerologie und Allergologie, Universitätsklinikum Schleswig-Holstein, Kiel, Deutschland

**Keywords:** Atopische Dermatitis, Versorgungsqualität, Lebensqualität, Patientenberichtete Endpunkte, Dermatologische Behandlung, Atopic dermatitis, Quality of health care, Quality of life, Patient-reported outcomes, Dermatological treatment

## Abstract

**Hintergrund:**

Mit AtopicHealth1 wurde 2010 die erste nationale Versorgungsstudie zur AD durchgeführt. Damals zeigte etwa ein Drittel der Patienten, die sich in einer Behandlung bei Dermatologen befanden, starke Einschränkungen der Lebensqualität, was auf eine unzureichende Versorgungsqualität hindeutete. Ziel der vorliegenden Studie war die Charakterisierung der aktuellen Versorgung von Patienten mit Neurodermitis in dermatologischer Behandlung im Schweregradvergleich sowie im Vergleich zu 2010 und zu Psoriasis.

**Methode:**

Die deutschlandweite multizentrische Querschnittstudie „AtopicHealth2“ erfasste klinische Daten, Lebensqualität (DLQI), Therapien, präventives Verhalten und patientendefinierten Behandlungsnutzen (PBI). Patienten mit einer Indikation für Systemtherapie wurden für Subgruppenanalysen als mittelschwer bis schwer, die anderen als leicht betroffen angesehen.

**Ergebnisse:**

Zwischen 2017 und 2019 wurden 1291 Patienten durch 111 Zentren eingeschlossen, mittleres Alter 41 Jahre, 56,5 % weiblich. Im Vergleich zu 2010 fanden sich keine Verbesserungen hinsichtlich Lebensqualität (jeweils DLQI 8,5), Schweregrad (SCORAD 45,4 vs. 42,3 in 2010) und Therapienutzen (PBI 2,2 vs. 2,4 in 2010). Mittelschwer bis schwer betroffene Patienten zeigten häufiger Lebensqualitätseinbuße (45,4 % vs. 23,6 %) und seltener relevante Therapienutzen (PBI < 1: 21,3 % vs. 13,2 %) als leichter betroffene. Verglichen mit Psoriasis offenbarten die Patienten mit Neurodermitis höhere Lebensqualitätseinschränkungen (DLQI 8,5 vs. 6,1) und einen geringeren Behandlungsnutzen (PBI 2,2 vs. 2,8).

**Diskussion:**

Im Vergleich zu 2010 zeigt sich keine verbesserte Versorgungsqualität von Neurodermitis in Deutschland. Im Vergleich zur Psoriasis weisen Patienten mit Neurodermitis höhere Belastungen und geringere therapeutische Nutzen auf, was den Bedarf an therapeutischen Innovationen unterstreicht.

## Abstract (en)

**Background:**

With AtopicHealth1, the first national care study on atopic dermatitis (AD) was conducted in 2010. At that time, about one third of the patients undergoing treatment by dermatologists showed severe limitations in quality of life, which indicated an insufficient quality of care. The aim of the present study was to characterise the current care of patients with AD undergoing dermatological treatment in comparison between different severity grades, as well as in comparison to 2010 and to psoriasis.

**Methods:**

The Germany-wide multicentre cross-sectional study “AtopicHealth2” recorded clinical data, quality of life (DLQI), therapies, preventive behaviour and patient-defined treatment benefit (PBI). Patients with an indication for systemic therapy were considered moderately to severely affected for subgroup analyses, the others mildly affected.

**Results:**

Between 2017 and 2019, 1291 patients (median age 41 years, 56.5% female) were enrolled by 111 centres. Compared with 2010, there were no improvements in quality of life (DLQI 8.5 in both studies), severity (SCORAD 45.4 vs. 42.3 in 2010) or treatment benefit (PBI 2.2 vs. 2.4 in 2010). Moderately to severely affected patients were more likely to show impaired quality of life (45.4% vs. 23.6%) and less likely to have relevant treatment benefits (PBI < 1: 21.3% vs. 13.2%) than mildly affected patients. In contrast to psoriasis, patients with AD revealed higher quality of life limitations (DLQI 8.5 vs. 6.1) and lower treatment benefit (PBI 2.2 vs. 2.8).

**Discussion:**

Compared to 2010, there is no improvement in the quality of care for AD in Germany. Compared to psoriasis, patients with AD show higher burden and lower treatment benefit, which underlines the need for therapeutic innovations.

Die Neurodermitis als eine der häufigsten Hautkrankheiten in Deutschland ist nach bisherigen Studien unterversorgt. Im vorliegenden Beitrag werden neue Daten zur Versorgungsqualität von Patienten mit Neurodermitis, die in dermatologischen Zentren behandelt werden, analysiert. Die Kenntnis der bestehenden Lücken könnte in Analogie zur Psoriasis, deren Versorgung sich inzwischen deutlich verbessert hat, in den nächsten Jahren eine Leitschiene für verbesserte Versorgungsqualität sein.

Die Neurodermitis oder atopische Dermatitis (AD) ist die häufigste chronische, rezidivierende, juckende und entzündliche Hauterkrankung, von der hauptsächlich Kinder, allerdings auch Erwachsene betroffen sind. In Deutschland hat die Neurodermitis bei Erwachsenen nach den aktuellsten Daten eine Einjahresbehandlungsprävalenz von 3,7 % und eine Punktprävalenz von 1,45 % [1, 2]. AD kann mit Lebensqualitätseinschränkungen [3, 4] und psychischen Belastungen [5, 6] einhergehen, sich auf die Schlafqualität [7] und die Arbeitsproduktivität auswirken und entsprechend sozioökonomische Folgen haben [8].

Mit AtopicHealth1 wurde 2010 die erste nationale Versorgungsstudie zur AD durchgeführt. Damals zeigte etwa ein Drittel der Patienten, die sich in einer Behandlung bei Dermatologen befanden, starke Einschränkungen der Lebensqualität und massive Schlafstörungen aufgrund der AD [9]. Bei unterstützenden Therapiemaßnahmen war die Basistherapie bei den meisten Patienten der Standard. In Bezug auf die durchgeführten Therapien konnte weitgehend von einer Versorgung ausgegangen werden, die den Empfehlungen der S2-Leitlinie der Deutschen Dermatologischen Gesellschaft (DDG), dem Berufsverband der Deutschen Dermatologen (BVDD) und anderen Fachgesellschaften und -gruppierungen [10–12] entsprach. Der Einsatz von topischen Glukokortikosteroiden und Calcineurininhibitoren stand dabei im Vordergrund. Die alltäglichen Belastungen durch die Erkrankung schienen jedoch bei einem Teil der Patienten in dermatologischer Behandlung erheblich zu sein, was darauf hindeuten könnte, dass in diesen Fällen therapeutische Optionen fehlten [9]. Im Vergleich zur Behandlung der Psoriasis (PSO), deren Versorgung sich durch die Einführung von innovativen Systemtherapeutika grundlegend geändert hat, gab es für Patienten mit AD seit Jahren kaum therapeutische Innovationen. Als neue Option in der Behandlung der mittelschweren bis schweren AD steht seit Oktober 2017 mit Dupilumab erstmals ein Biologikum zur Verfügung.

Im Mittelpunkt der vorliegenden Studie AtopicHealth2 steht die Charakterisierung der Versorgungsqualität von Patienten mit Neurodermitis in dermatologischer Behandlung, die im Schweregradvergleich sowie im Vergleich zu 2010 und zu Psoriasis erfolgen soll.

## Patienten und Methoden

### Studiendesign

Es handelt sich um eine nichtinterventionelle, multizentrische Querschnittstudie. Die Hälfte der teilnehmenden Dermatologen wurde gebeten, keine Vorauswahl zu treffen und Patienten mit allen Schweregraden konsekutiv einzuschließen. Diese Erhebungsweise entspricht der vorangegangenen Studie AtopicHealth1 [9]. Die andere Hälfte der Dermatologen sollte nur Patienten mit einer Indikation für Systemtherapie (ST) einschließen. Patienten mit einer Indikation für ST wurden als mittelschwer bis schwer von AD betroffen eingeordnet. Im Anschreiben wurden die betreffenden Dermatologen gebeten, anhand der folgenden Definition über einen Einschluss zu entscheiden: *„Eine mittelschwere bis schwere Neurodermitis liegt grundsätzlich dann vor, wenn der Patient für eine Systemtherapie geeignet ist (diese muss aber nicht unmittelbar durchgeführt werden).“*

Die AtopicHealth-Reihe lehnt sich an die nationale Studienreihe PsoHealth zur PSO [13] an und, wenn möglich, wurden identische Ergebnisparameter erhoben. Im vorliegenden Artikel wird die Versorgungssituation von Patienten mit AD 2017 bis 2019 bei verschiedenen Schweregraden mit der vorausgegangenen Studie der AtopicHealth-Reihe, AtopicHealth1 (2010) [9], und der neuesten Studie der PsoHealth-Reihe, PsoHealth4 (2016/17) [14], verglichen.

### Rekrutierung

Die Rekrutierung der Patienten fand in dermatologischen Praxen und Kliniken statt. Die Auswahl der teilnehmenden Zentren erfolgte mittels Ziehung einer Zufallsstichprobe aus einer Liste mit allen in Deutschland tätigen Dermatologen, die Mitglied im BVDD sind. Es wurden 1692 Zentren kontaktiert; davon erklärten sich 190 (11,2 %) zur Teilnahme bereit. Einhundertelf Zentren beteiligten sich deutschlandweit durch Patienteneinschlüsse. Der Anteil von teilnehmenden Kliniken war vergleichbar zur Neurodermitis-Studie 2010 und etwas höher als in PsoHealth4 (Tab. 1). Eingeschlossen wurden in allen 3 Studien Patienten ab 18 Jahren, die eine Einwilligungserklärung unterschrieben hatten. Vor Beginn der Studien wurde die Zustimmung der zuständigen Ethikkommission eingeholt.

**Table 1:** 

		AtopicHealth2 (*n* = 1291 Patienten)		AtopicHealth1 (*n* = 1678 Patienten)		PsoHealth4 (*n* = 1827 Patienten)	
		*n*	*%*	*n*	*%*	*n*	*%*
Anteil Praxen/Kliniken (Ambulanzen) an aktiven Zentren	Praxen	96	86,5	79	86,8	84	90,3
	Kliniken	15	13,5	12	13,2	9	9,7
Anteil Einschlüsse durch Praxen/Kliniken (Ambulanzen)	Praxen	1053	81,6	1217	72,5	1661	90,9
	Kliniken	237	18,4	461	27,5	166	9,1
		*MW* *±* *SD*	*Median*	*MW* *±* *SD*	*Median*	*MW* *±* *SD*	*Median*
Einschlüsse pro Zentrum	Alle Zentren	11,6 ± 8,9	9,0	18,4 ± 21,5	12,0	17,8 ± 15,0	15,0

### Erfasste Daten in AtopicHealth2

#### A. Arztfragebogen

Erhoben wurden klinische Merkmale der AD, Begleiterkrankungen, der Schweregrad der AD (SCORing Atopic Dermatitis [SCORAD] [15]) sowie bisherige Therapien.

#### B. Patientenfragebogen

Erfragt wurden soziodemografische Aspekte, die hauterkrankungsbezogene Lebensqualität (Dermatology Life Quality Index [DLQI] [16]), der Schweregrad der AD-Symptome (Patient Oriented Eczema Measure [POEM] [17]), pruritusbezogene Belastungen (Einzelitems, übernommen aus AtopicHealth1, in Anlehnung an den Fragebogen zur Messung der Lebensqualität bei Patienten mit chronischem Pruritus [ItchyQoL]), der selbst eingeschätzte Gesundheitszustand (European Quality of Life – Visuelle Analogskala [EQ VAS] [18]), die Ausdehnung der Neurodermitisläsionen in Anlehnung an die Methode von Bahmer et al. [19] (body surface area [BSA]), Therapien der letzten 5 Jahre, präventives Verhalten, Bestandteile des Anamnesegesprächs und der patientendefinierte Behandlungsnutzen (Patient Benefit Index [PBI] [20]). Parameter, die als Versorgungsqualitätsindikatoren herangezogen wurden, werden nachfolgend beschrieben.

### Indikatoren für Versorgungsqualität

Die Indikatoren zur Versorgungsqualität der Neurodermitis, die ein Expertenkonsensus entwickelt hatte, wurden teilweise bereits auf die Studie AtopicHealth1 angewendet [21]. Dabei wurde zwischen Ergebnis- und Prozessqualitätsindikatoren unterschieden (Tab. 2).

**Table 2:** 

Indikatoren der Prozessqualität		Arztfragebogen (AtopicHealth2)	Patientenfragebogen (AtopicHealth2)
Anamnese	Erfassung der Provokationsfaktoren der AD durch den Dermatologen	–	x
	Erfragung der atopischen Krankheitsgeschichte durch den Dermatologen	–	x
Diagnostik	Anwendung der Kriterien nach Hanifin und Rajka [22]	–	x
	Durchführung des RAST = Phadiatop-Tests	x	–
Therapie	Regelmäßige Hautpflege	–	x
	Einsatz von topischen Steroiden oder Immunmodulatoren	–	x
Prävention	Vermeidung von Provokationsfaktoren	–	x
	Durchführung einer Hausstaubsanierung (nur bei Hausstauballergikern)	–	x
	Verzicht auf das Rauchen in der eigenen Wohnung	–	x
	Individuelle Beratung beim Facharzt	–	x
	Teilnahme an Neurodermitisschulungen	–	x
*Indikatoren der Ergebnisqualität*			
Lebensqualität	Mittlerer DLQI	–	x
	Anteil der Patienten mit hohen Lebensqualitätseinschränkungen (DLQI > 10)	–	x
Schweregrad der AD	Mittlerer Schweregrad (SCORAD)	x	–
	Anteil der Patienten mit hohem Schweregrad (SCORAD ≥ 50)	x	–
Kein minimaler Therapienutzen	Patientennutzen-Index < 1	–	x

*AD* atopische Dermatitis/Neurodermitis, *DLQI* Dermatology Life Quality Index, *RAST* Radio-Allergo-Sorbent-Test, *SCORAD* SCORing Atopic Dermatitis

### Versorgungsqualität im Vergleich zu Psoriasis

Auch für die Versorgung der PSO wurden im Expertenkonsensus Versorgungsqualitätsindikatoren entwickelt [23]. Die nicht erkrankungsspezifischen Indikatoren wurden im Rahmen dieser Studie zwischen AD und PSO verglichen:

- Lebensqualität (mittlerer DLQI und Anteil [%] DLQI > 10),
- systemische Therapie in den letzten 5 Jahren (% der Patienten),
- stationäre Behandlung in den letzten 5 Jahren (% der Patienten),
- patientendefinierter Therapienutzen (mittlerer PBI).

### Statistische Auswertung

Die Daten wurden deskriptiv ausgewertet und mit statistischen Standardmaßen beschrieben (absolute und relative Häufigkeiten bei kategorialen Daten; Mittelwert und Standardabweichung bei kontinuierlichen Daten).

**Figure 1:**
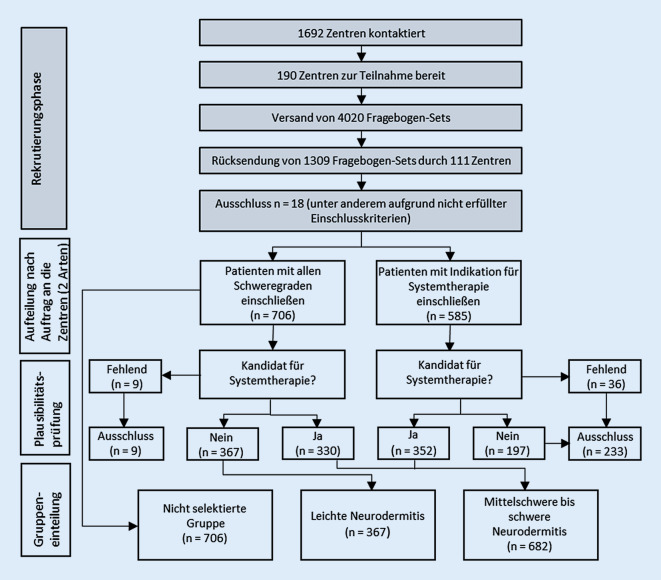

Für die folgenden Auswertungen lassen sich 3 Gruppen unterscheiden: a) Patienten ohne schweregradbezogene Vorauswahl, b) Patienten mit Indikation für ST, die entweder aus der Gruppe ohne Vorauswahl oder der Gruppe mit Vorauswahl nach Indikation für ST stammten, und c) Patienten ohne Indikation für ST (Abb. 1). Entsprechend gibt es eine Überschneidung von Gruppe a) mit b) und c), weshalb nur b) und c) direkt miteinander verglichen werden sollten und a) für einen Vergleich mit den Studien AtopicHealth1 und PsoHealth4 verwendet werden sollte, bei denen ebenfalls keine Vorauswahl getroffen wurde.

Häufigkeitsunterschiede wurden mithilfe von Chi-Quadrat-Tests analysiert, Mittelwertunterschiede durch t‑Tests für unabhängige Stichproben. Die Ergebnisse der Signifikanztests wurden um Effektgrößen ergänzt (Cohens d bei t‑Tests und Phi-Koeffizienten [φ] bei Chi-Quadrat-Tests). Laut Cohen steht ein d von 0,2, 0,5 und 0,8 für schwache, mittlere und starke Effekte bei t‑Tests für unabhängige Stichproben [24]. Bei Phi-Koeffizienten werden die Effektgrößen von 0,1, 0,3 und 0,5 nach Cohen als klein, mittel und groß betrachtet [24]. In einer Post-hoc-Poweranalyse für t‑Tests für unabhängige Stichproben, bei der wir uns am Effekt des DLQI-Mittelwertunterschiedes im Schweregradvergleich (vgl. Tab. 6) orientiert haben, wurde bei zweiseitiger Testung, einer Effektgröße d von 0,5, einem Alpha-Wert von 0,05 und Stichprobengrößen von 682 und 376 eine Power von 1,0 erzielt.

Unterschiede mit einer Wahrscheinlichkeit eines Alpha-Fehlers von < 0,05 wurden als statistisch signifikant betrachtet. Fehlende Werte wurden nicht ersetzt. Die Auswertung erfolgte mit SPSS 26 (IBM, Armonk, NY, USA) für Windows.

## Ergebnisse

### Zentren und Patienten

Von den 4020 verschickten Fragebogensets (Arzt- und Patientenbogen) wurden 1309 Exemplare zwischen August 2017 und Juni 2019 zurückgesendet. Es wurden 18 Fragebogensets ausgeschlossen (*n* = 1: Einschlusskriterien nicht erfüllt; *n* = 4: widersprüchliche Aussagen in Arzt- und Patientenfragebogen z. B. zum Alter, die auf unterschiedliche Personen hindeuteten; *n* = 13: unvollständiges Set, da Arzt- oder Patientenbogen nicht ausgefüllt). Damit ergab sich eine Gesamtzahl von 1291 Datensätzen. Darunter stammten 706 Patienten aus Zentren, die keine schweregradbezogene Vorauswahl treffen sollten, und 585 aus Zentren, die gezielt Patienten mit Indikation für eine ST einschließen sollten (Abb. 1). Bei 682 Patienten wurde nach Prüfung auf Plausibilität eine Indikation für ST angenommen, bei 367 war dies nicht der Fall.

### Soziodemografische und klinische Merkmale

Unter den Patienten der Gruppe ohne Vorselektion waren 55,8 % weiblich. Das mittlere Alter lag bei 41 Jahren. Der größte Teil hatte ein Abitur und war erwerbstätig (Tab. 3).

**Table 3:** 

		Ohne Vorselektion (*n* = 706^a^)		Indikation für ST (*n* = 682^a^)		*Keine* Indikation für ST (*n* = 367^a^)	
		*n*	%	*n*	%	*n*	%
Geschlecht	Männlich	301	44,2	347	53,0	118	33,2
	Weiblich	380	55,8	308	47,0	237	66,8
Altersgruppen	18 bis 30 Jahre	221	35,1	158	25,9	136	41,8
	> 30 bis 50 Jahre	235	37,4	253	41,5	108	33,2
	> 50 bis 70 Jahre	140	22,3	170	27,9	66	20,3
	> 70 Jahre	33	5,2	28	4,6	15	4,6
Schulabschluss	(Fach‑)Abitur	329	49,3	286	44,0	182	52,0
	Realschule	236	35,4	242	37,2	127	36,0
	Hauptschule	102	15,3	122	18,8	40	11,0
Beruflicher Status	Erwerbstätig	479	68,6	473	70,7	244	67,0
	Erwerbslos/arbeitssuchend	11	1,6	14	2,1	7	1,9
	Altersrente	80	11,5	75	11,2	37	10,2

*ST* Systemtherapie

^a^Fehlende Angaben variieren

Rhinoconjunctivitis allergica und Asthma bronchiale waren die häufigsten Begleiterkrankungen. Patienten mit einer Indikation für ST berichteten häufiger von pruritusbedingten Schlafstörungen, zeigten einen höheren Schweregrad, gaben einen höheren Anteil betroffener Körperoberfläche an und bewerteten ihren Gesundheitszustand negativer (Tab. 4).

**Table 4:** 

		Indikation für ST (*n* = 682^a^)		Keine Indikation für ST (*n* = 367^a^)		χ^2^	df	*p*	φ^b^
		*n*	%	*n*	%				
Schlafstörungen wegen Juckreiz	Nie bis manchmal	389	57,6	285	77,7	41,7	1	< 0,001	0,2
	Häufig bis immer	286	42,4	82	22,3				
Allergische Begleiterkrankungen	Allergisches Asthma bronchiale	251	37,2	85	23,4	20,8	1	< 0,001	0,1
	Rhinoconjunctivitis allergica	333	49,4	151	41,5	5,9	1	< 0,05	0,1
	Kontaktallergie	112	16,6	43	11,8	4,3	1	< 0,05	0,1
	Urtikaria/Angioödem	25	3,7	10	2,7	0,7	1	0,41	0,0
	**Möglicher Range**	***n***	**MW** **±** **SD**	***n***	**MW** **±** **SD**	**t**	**df**	***p***	**d**^b^
Betroffene Körperoberfläche (BSA)	0 bis 100 %	659	21,5 ± 22,0	363	8,6 ± 10,9	−12,5	1010,6	< 0,001	0,7
Symptomschwere (POEM)	0 bis 28	670	17,9 ± 7,2	360	13,7 ± 7,0	-8,9	1028	< 0,001	0,6
Gesundheitszustand (EQ VAS)	0 = schlechtest denkbarer bis 100 = best denkbarer	667	57,4 ± 23,0	358	68,4 ± 19,6	8,1	833,5	< 0,001	0,5
Jahre seit Diagnose	0 bis aktuelles Lebensalter	607	31,0 ± 16,2	323	24,1 ± 15,7	−6,3	928	< 0,001	0,4

*BSA* body surface area, *EQ VAS* European Quality of Life Visual Analogue Scale, *POEM* Patient Oriented Eczema Measure, *ST* Systemtherapie

^a^Fehlende Angaben variieren

^b^Effektgröße

### Merkmale der Versorgung

Nach Arztangaben hatten 90,1 % der Patienten (Gruppe ohne Vorselektion) jemals topische Therapien verwendet. Phototherapien wurden am zweithäufigsten angegeben (46,6 %). ST wurde von 35,3 % jemals angewendet, darunter am häufigsten systemische Glukokortikosteroide (27,4 %). Nach Angabe der Patienten hatten 43,0 % in den letzten 5 Jahren eine ST erhalten.

### Prozessindikatoren der Versorgungsqualität

#### Ärztliche Anamnese und Diagnostik

Die meisten Patienten waren sich sicher, im Arztkontakt jemals nach Juckreiz und den betroffenen Körperregionen gefragt worden zu sein. Am seltensten wurde angegeben, nach Auslösefaktoren gefragt worden zu sein (Tab. 5). Diejenigen mit Indikation für ST gaben häufiger an, gefragt worden zu sein, ob noch andere atopische Erkrankungen vorlägen und ob die AD chronisch sei.

**Table 5:** 

			Indikation für ST (*n* = 682^a^)		*Keine* Indikation für ST (*n* = 367^a^)		χ^2b^	df	*p*	φ
			*n*	%	*n*	%				
*Anamnese: Wurde im Arztkontakt jemals erfragt/untersucht, ob*										
… es Auslöser für Ihre AD gibt?	Ja (unsicher)		419 (105)	62,0 (15,5)	204 (60)	56,8 (16,7)	2,6	1	0,109	0,1
… Sie noch andere atopische Erkrankungen haben?	Ja (unsicher)		571 (33)	84,5 (4,9)	278 (21)	77,2 (5,8)	8,8	1	< 0,01	0,1
… bei Ihnen Juckreiz vorliegt?	Ja (unsicher)		599 (22)	88,7 (3,3)	305 (12)	85,0 (3,3)	3,8	1	0,050	0,1
… Sie die AD im Gesicht, Kopfhaut, Hände, Armbeugen und/oder Kniekehlen haben?	Ja (unsicher)		603 (19)	89,1 (2,8)	315 (12)	86,3 (3,3)	1,6	1	0,207	0,0
… die AD chronisch oder wiederkehrend ist?	Ja (unsicher)		518 (48)	76,7 (7,1)	248 (37)	69,1 (10,3)	4,3	1	< 0,05	0,1
… Familienmitglieder von atopischen Erkrankungen betroffen sind?	Ja (unsicher)		530 (40)	78,1 (5,9)	267 (23)	73,4 (6,3)	3,2	1	0,076	0,1
*Diagnostik*										
Phadiatop-Test (Sx1) durchgeführt?	Ja		106	18,5	58	18,4	0,0	1	0,967	0,0
Alle Kriterien nach Hanifin & Rajka angewendet?	Ja		401	60,6	175	50,0	10,4	1	< 0,01	0,1
*Therapie*										
Topische Steroide oder Immunmodulatoren in den letzten 5 Jahren?	Ja		611	90,3	314	86,0	4,2	1	< 0,05	0,1
Regelmäßige Hautpflege durchgeführt?	Ja (teilweise)		626 (46)	92,6 (6,8)	327 (35)	89,8 (9,6)	0,0	1	0,960	0,0
*Präventionsmaßnahmen*										
Meiden von Auslösefaktoren?	Meidung hautreizender Mittel	Ja (teilweise)	542 (86)	81,3 (12,9)	255 (65)	70,2 (17,9)	13,9	1	< 0,001	0,1
	Verzicht auf Haustiere	Ja (teilweise)	422 (29)	64,8 (4,5)	199 (11)	57,7 (3,2)	6,5	1	< 0,05	0,1
	Vermeidung bestimmter Nahrungsmittel	Ja (teilweise)	379 (94)	57,0 (14,1)	168 (49)	47,9 (14,0)	9,7	1	< 0,01	0,1
Hausstaubsanierung bei Hausstauballergikern?	Ja (teilweise)		284 (70)	69,6 (17,2)	117 (28)	67,6 (16,2)	0,8	1	0,371	0,0
Auf Rauchen in der eigenen Wohnung verzichtet?	Ja (teilweise)		504 (20)	80,4 (3,2)	253 (9)	76,4 (2,7)	2,8	1	0,096	0,1
Persönliche Beratung über die Erkrankung bei einem Facharzt erfolgt?	Ja (teilweise)		549 (43)	81,9 (6,4)	260 (47)	73,0 (13,2)	2,1	1	0,151	0,1
An mindestens einer AD-Schulung teilgenommen?	Ja (teilweise)		185 (46)	28,1 (7,0)	58 (22)	16,7 (6,3)	16,8	1	< 0,001	0,1

*AD* atopische Dermatitis/Neurodermitis, *ST* Systemtherapie

^a^Fehlende Angaben variieren

^b^Ausschluss der Angaben „unsicher“ und „teilweise“ bei Test auf Häufigkeitsunterschiede

#### Therapeutische und präventive Maßnahmen

Fast alle Patienten gaben an, regelmäßige Hautpflege durchzuführen sowie in den letzten 5 Jahren topische Steroide oder Immunmodulatoren angewendet zu haben. Patienten mit einer Indikation für ST gaben häufiger an, eine Neurodermitisschulung besucht zu haben, hautreizende Mittel und bestimmte Nahrungsmittel zu meiden sowie auf Haustiere zu verzichten.

### Ergebnisindikatoren der Versorgungsqualität

Die Patienten mit einer Indikation für eine ST zeigten einen höheren Schweregrad (SCORAD) und eine geringere Lebensqualität (DLQI) als diejenigen ohne Indikation für ST. Patienten mit Indikation für ST erfuhren häufiger einen unzureichenden Therapienutzen (PBI < 1) (Tab. 6).

**Table 6:** 

		Indikation für ST		*Keine* Indikation für ST		t	df	*p*	d
	Möglicher Range	*n*	MW ± SD	*n*	MW ± SD				
Mittlere Lebensqualität (DLQI)	0 (keine) bis 30 (max. LQ-Einschränkung)	678	10,7 ± 7,0	364	7,4 ± 5,8	-8,1	867,2	< 0,001	0,5
Mittlerer Schweregrad (SCORAD)	0 (keine Betroffenheit) bis 103 (max. Schweregrad)	635	57,2 ± 18,9	351	38,3 ± 17,3	-15,5	984	< 0,001	1,0
	*Kriterium*	*n*	*%*	*n*	*%*	*χ*^*2*^	*df*	*p*	*φ*
Starke Lebensqualitätseinschränkungen	Anteil Patienten mit DLQI > 10	308	45,4	86	23,6	47,8	1	< 0,001	0,2
Hoher klinischer Schweregrad	Anteil Patienten mit SCORAD ≥ 50	436	68,7	88	25,1	172,5	1	< 0,001	0,4
Kein Therapienutzen	Anteil Patienten mit PBI < 1	138	21,3	45	13,2	9,7	1	< 0,01	0,1

*LQ* Lebensqualität, *DLQI* Dermatology Life Quality Index, *SCORAD* SCORing Atopic Dermatitis, *ST* Systemtherapie, *PBI* Patient Benefit Index

### Versorgungsqualität im Vergleich zu AtopicHealth1 (2010)

**Figure 2:**
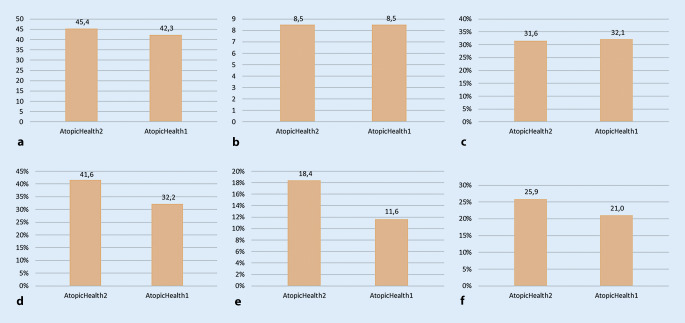

Der mittlere SCORAD in der Studie AtopicHealth2 (nur Patienten ohne Vorselektion, s. Kapitel „statistische Auswertung“) von 45,4 ± 20,6 war höher als in AtopicHealth1 (t(1146,4) = 3,4, *p* < 0,01, d = 0,2; Abb. 2), und häufiger wurde ein SCORAD ≥ 50 berichtet (χ^2^(1) = 18,4, *p* < 0,001, φ = 0,1). Der DLQI lag im Mittel bei 8,5 ± 6,5 (DLQI > 10: 31,6 %). Er unterschied sich nicht von AtopicHealth1. Der patientendefinierte Therapienutzen (PBI) war niedriger als in AtopicHealth1 (2,2 ± 1,1 vs. 2,4 ± 1,1, t(1252) = 4,2, *p* < 0,001, d = 0,2) und häufiger wurde von Krankenhausaufenthalten berichtet (χ^2^(1) = 6,2, *p* < 0,05, φ = 0,1; Abb. 2). Bei einer separaten Betrachtung von nur solchen Patienten, die durch Kliniken eingeschlossen wurden, zeigten sich keine Unterschiede in diesen Parametern zwischen den beiden Studien.

### Versorgungsqualität im Vergleich zu Psoriasis

**Figure 3:**
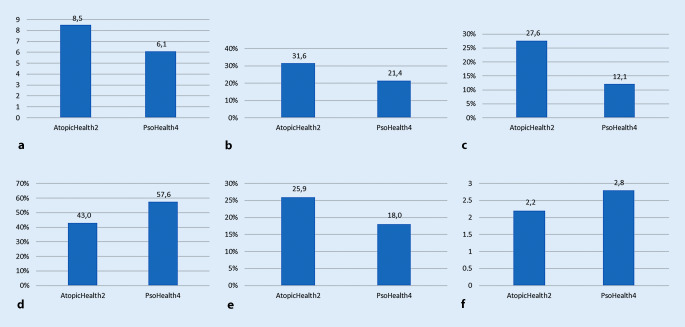

In vergleichbaren Versorgungsqualitätsindikatoren zeigten Patienten mit AD höhere Lebensqualitätseinschränkungen als Patienten mit PSO (t(2464) = 8,2, *p* < 0,001, d = 0,4) und häufiger einen DLQI > 10 (χ^2^(1) = 28,7, *p* < 0,001, φ = 0,1). Mehr Patienten mit PSO hatten in den letzten 5 Jahren mindestens einmal ST erhalten (χ^2^(1) = 42,6, *p* < 0,001, φ = 0,1). Der Anteil mit Krankenhausaufenthalten in den letzten 5 Jahren war bei Patienten mit AD etwas höher (χ^2^(1) = 17,5, *p* < 0,001, φ = 0,1), und der patientendefinierte Therapienutzen war bei ihnen deutlich niedriger als bei Patienten mit PSO (t(1861) = 10,4, *p* < 0,001, d = 0,5; Abb. 3). Bei einer separaten Betrachtung von nur solchen Patienten, die durch Kliniken eingeschlossen wurden, zeigte sich kein Unterschied im Anteil an Patienten mit Krankenhausaufenthalten und Systemtherapie zwischen den beiden Studien.

### Diskussion

Das Ziel der vorliegenden Studie AtopicHealth2 war es, die Versorgungsqualität der Neurodermitis in Deutschland zu charakterisieren. Hierzu wurden bundesweit Daten von Patienten und ihren Ärzten in dermatologischen Praxen und Ambulanzen gewonnen. Die Methodik lehnt sich an eine vorausgehende Erhebung aus dem Jahr 2010 an. Untersucht wurden sowohl klinische Ergebnisindikatoren wie auch Prozessindikatoren. Es sollte ferner untersucht werden, ob es Anhaltspunkte für Unterschiede in der Versorgung von Patienten mit leichter vs. mittelschwerer bis schwerer AD gibt, ob sich die Versorgung im Vergleich zu 2010 verändert hat und ob sich die Versorgungsqualität der AD von der der PSO unterscheidet.

Ein beträchtlicher Anteil von Patienten zeigte einen hohen klinischen Schweregrad der AD, gemessen am SCORAD (Mittelwert, MW = 45,4) und BSA (MW = 14,3 %). Auch die patientenberichteten Einschränkungen und damit die Krankheitsbelastung waren vergleichsweise hoch, gemessen am POEM (MW = 15,3) und am DLQI (MW = 8,5). Wie bereits in der vorausgehenden Studie von 2010 [9] beschreibt auch in der aktuellen Studie etwa ein Drittel der Patienten eine erhebliche Beeinträchtigung der Lebensqualität, was angesichts eines hohen Anteils an Patienten unter laufender Therapie beachtenswert ist. Die häufige Betroffenheit von reduzierter Schlafqualität und starkem Juckreiz unterstreicht das hohe Ausmaß an Beeinträchtigungen der Patienten und damit oftmals unzureichende Behandlungsergebnisse. Insgesamt ist das Ausmaß der hier untersuchten Krankheitsbelastungen 2017 bis 2019 nicht geringer als 2010. Ein Grund könnte in fehlenden Fortschritten der Arzneimittelversorgung liegen, die sich in vorausgehenden Studien bei Psoriasis als zeitlich koinzidierende Größe erwiesen hat.

Die Patienten mit Indikation für eine ST zeigen höhere klinische und subjektive Belastungen und berichten häufiger, dass sie keinen Therapienutzen haben. Aber auch unter den Patienten ohne Indikation für eine ST weist etwa ein Viertel erhebliche Einbußen der Lebensqualität auf, was zeigt, dass auch aus klinischer Sicht leicht betroffene Patienten starke Beeinträchtigungen erleben, die offenbar durch die derzeitige Behandlung nicht angemessen reduziert werden.

Wie schon in der Vorgängerstudie 2010 gibt es einen hohen Anteil von Patienten, die topische Steroide und/oder Immunmodulatoren anwenden und präventive Maßnahmen wie regelmäßige Hautpflege durchführen, was vordergründig einer leitliniengerechten Behandlung entspricht. Dennoch findet sich auch in der aktuellen Studie ein vergleichsweise geringer Behandlungsnutzen.

Vor allem im Vergleich zu Patienten mit PSO zeigen sich Unterschiede. Die Versorgungsqualität, gemessen anhand vordefinierter Kriterien, hat sich bei PSO [13], nicht aber bei AD über die Zeit verbessert. Dies weist auf eine weiterhin hohe Notwendigkeit von qualitätsverbessernden Maßnahmen in der Versorgung der AD hin. Die ständig wachsende Anzahl an Biologika und weiteren innovativen Systemtherapeutika bei PSO sowie deren zunehmende Effektivität haben dazu beigetraten, dass im Zuge der erweiterten technologischen Möglichkeiten eine bessere Versorgungsqualität erreicht werden konnte. Bisher war dies für AD nicht der Fall, denn bis 2017 gelangten keine entsprechenden Innovationen in die Versorgung [25]. Dies hat sich inzwischen mit Dupilumab geändert; der Januskinaseinhibitor Baricitinib wurde im September 2020 zur Therapie der AD von der European Medicines Agency (EMA) zugelassen, und weitere Innovationen werden in den nächsten Jahren folgen. Bei der Angabe ihrer aktuellen Therapie hatten Patienten teilweise freitextlich ergänzt, dass sie Dupilumab im Rahmen einer Studie erhalten würden. Das Medikament aktuell zu erhalten wurde von 12,8 % der Patienten angegeben. Dies waren bis auf 2 Patienten solche, bei denen der Arzt auch eine Indikation für ST angegeben hatte. Der vergleichsweise hohe Anteil an Patienten mit Dupilumab-Exposition könnte an der Beteiligung von überproportional vielen forschenden dermatologischen Praxen und Ambulanzen gelegen haben, was die Repräsentativität einschränken würde.

Bei der Interpretation der Indikatoren zur Prozessqualität ist zu beachten, dass sie von den Patienten selbst beurteilt und erinnert wurden und diese das Abfragen diagnostischer Kriterien und weiterer Prozessmerkmale im Arztkontakt möglicherweise anders verstanden haben könnten. Außerdem ist ein Recall Bias wie bei allen retrospektiv erhobenen Daten nicht auszuschließen.

Eine weitere Einschränkung der vorliegenden Studie ist die Möglichkeit von Zentrums- und Patientenselektionen aufgrund der Freiwilligkeit der Teilnahme. Dieses Phänomen kann in der Versorgungsforschung unter Routinebedingungen nicht verhindert werden, sodass die Aussagen der Studie im Wesentlichen auf Patienten und Praxen mit hoher Teilnahmebereitschaft beruhen.

In der vorliegenden Studie wurden nur Patienten in dermatologischer Versorgung beobachtet. Patienten, die von anderen Facharztgruppen versorgt werden, und Patienten ohne ärztliche Behandlung fanden keine Berücksichtigung. Dermatologen stellen in Deutschland einen relevanten Anteil der Versorger dar, wobei man davon ausgehen kann, dass die so versorgten Patienten einer tendenziell schwerer betroffenen Gruppe angehören. Deshalb sollten weitergehende Studien auch in allgemeinmedizinischen und pädiatrischen Zentren durchgeführt werden, in denen möglicherweise Patienten mit anderen Merkmalen anzutreffen sind. In einer Studie mit Mitgliedern einer deutschen Neurodermitis-Selbsthilfeorganisation, deren Versorgungsprofil sehr heterogen war (inklusive solcher ohne ärztliche Versorgung), fand sich dieselbe Lebensqualitätseinschränkung (DLQI) wie in beiden AtopicHealth-Studien [26]. Zusammengefasst stellt die vorliegende Studie einen wichtigen Meilenstein in der Forschung zur Versorgungsqualität bei Neurodermitis in Deutschland dar, der auf hohen Handlungsbedarf in der Versorgung und die Notwendigkeit des Einsatzes innovativer Behandlungskonzepte hinweist.

## Fazit für die Praxis

- Im Vergleich zu 2010 hat sich die Versorgungsqualität von Patienten mit Neurodermitis in dermatologischer Behandlung bis 2019 nicht verbessert.
- Die häufige Betroffenheit von reduzierter Schlafqualität und starkem Juckreiz deutet auf hohe Beeinträchtigungen der Patienten und oftmals unzureichende Behandlungsergebnisse hin.
- Patienten mit einem höheren Schweregrad der Neurodermitis berichten häufiger, dass sie keinen Therapienutzen haben.
- Im Vergleich zur Psoriasis zeigen Patienten mit Neurodermitis höhere Belastungen und geringere therapeutische Nutzen.
- Die Implementierung neuer Wirkstoffe sollte fokussiert werden, um in Analogie zur Psoriasis die Lebensqualität der Patienten nachhaltig zu bessern.
